# Untangling the Effects of Plant Genotype and Soil Conditions on the Assembly of Bacterial and Fungal Communities in the Rhizosphere of the Wild Andean Blueberry (*Vaccinium floribundum* Kunth)

**DOI:** 10.3390/microorganisms11020399

**Published:** 2023-02-04

**Authors:** Dario X. Ramirez-Villacis, Andrea Pinos-Leon, Pamela Vega-Polo, Isai Salas-González, Corbin D. Jones, Maria de Lourdes Torres

**Affiliations:** 1 Laboratorio de Biotecnología Agrícola y de Alimentos—Ingeniería en Agronomía, Politécnico, Universidad San Francisco de Quito (USFQ), Quito 170901, Ecuador; 2 Instituto de Microbiología, Universidad San Francisco de Quito (USFQ), Quito 170901, Ecuador; 3 Department of Biology, University of North Carolina at Chapel Hill, Chapel Hill, NC 27514, USA; 4 Laboratorio de Biotecnología Vegetal, Colegio de Ciencias Biológicas y Ambientales, Universidad San Francisco de Quito (USFQ), Quito 170901, Ecuador; 5 Curriculum in Bioinformatics and Computational Biology, University of North Carolina at Chapel Hill, Chapel Hill, NC 27514, USA

**Keywords:** Ecuadorian Highland, edaphic factors, microbiome, plant genotype, rhizosphere, *Vaccinium floribundum*

## Abstract

Microbial communities in the rhizosphere influence nutrient acquisition and stress tolerance. How abiotic and biotic factors impact the plant microbiome in the wild has not been thoroughly addressed. We studied how plant genotype and soil affect the rhizosphere microbiome of *Vaccinium floribundum*, an endemic species of the Andean region that has not been domesticated or cultivated. Using high-throughput sequencing of the 16S rRNA and ITS region, we characterized 39 rhizosphere samples of *V. floribundum* from four plant genetic clusters in two soil regions from the Ecuadorian Highlands. Our results showed that Proteobacteria and Acidobacteria were the most abundant bacterial phyla and that fungal communities were not dominated by any specific taxa. Soil region was the main predictor for bacterial alpha diversity, phosphorous and lead being the most interesting edaphic factors explaining this diversity. The interaction of plant genotype and altitude was the most significant factor associated with fungal diversity. This study highlights how different factors govern the assembly of the rhizosphere microbiome of a wild plant. Bacterial communities depend more on the soil and its mineral content, while plant genetics influence the fungal community makeup. Our work illustrates plant–microbe associations and the drivers of their variation in a unique unexplored ecosystem from the Ecuadorian Andes.

## 1. Introduction

Plants and their root-associated microorganisms have developed symbiotic relationships that help them adapt to environmental changes [1]. In the narrow zone around the plant root called the rhizosphere, the interactions between the host with the bacteria and fungi present in the soil are complex due to environmental factors, both abiotic and biotic [1,2]. As the plant’s microbiome can enhance stress tolerance, disease resistance, and nutrient uptake, understanding the factors that govern the recruitment and assembly of the rhizosphere communities is key to comprehending plant evolution and improving production yield [1].

Most of the studies on microbiome assembly have focused on crop species and the model plant *Arabidopsis thaliana* [3]. These studies showed that the abiotic factors, notably soil properties such as nutrients and pH, affect the microbial communities directly by affecting the growth of the microorganisms, and indirectly by influencing the plant physiology and root exudation patterns [4,5,6]. In addition, plant genotype has been reported as the main biotic factor that influences the root microbiome assemblages by producing different hormones and exudates, causing selective pressure on bacterial and fungal communities [2,7,8]. For instance, different genotypes of the same plant species are associated with distinct traits, root architecture, growth rates, and physiological processes that shape the rhizosphere microbiome assembly [9,10].

However, studies of rhizosphere communities and the factors affecting microbiome assembly in wild plant species in their native environments are scarce, especially in highland ecosystems such as the Andes [10,11,12,13,14]. Therefore, our goal is to establish the effect of soil (i.e., abiotic factor) and plant genotype (i.e., biotic factor) in the composition and diversity of the bacterial and fungal communities in the rhizosphere microbiome of the Andean blueberry (*Vaccinium floribundum* Kunth.) in its native environment.

We chose *V. floribundum* for various reasons. First, this plant species is a shrub endemic to the Andean region (from Venezuela to Bolivia) [15] and is found in the *páramo,* a unique Neotropical ecosystem characterized by its non-arboreal vegetation, soil acidity, high ultraviolet irradiation, low temperatures, and high humidity [16,17]. Second, its fruits are known for their nutritional functions due to their high antioxidant capacity and high content of anthocyanins and polyphenolic compounds [16]. In addition, *V. floribundum* has a high cultural value in Ecuador since its berries are used mainly by rural communities for ritual purposes and the preparation of traditional food [16,17]. A fourth reason is that this species has not been domesticated or cultivated [16], which makes experiments at the laboratory level or in control conditions practically impossible. Finally, the population structure of *V. floribundum* in the Ecuadorian Highlands has been described, and the genetic information (simple sequence repeats SSRs) is publicly available [18].

For this natural experiment, we collected rhizosphere samples from *V. floribundum* individuals from the four plant genetic clusters previously reported [18] along two different soil regions in the Ecuadorian Highlands [19]. We found that the soil region significantly affects bacteria diversity, while the interaction of plant genotype and altitude influences fungal community assembly. Going deeper, two specific soil elements (phosphorus and lead) could recapitulate the effect observed by the soil region on bacterial communities. Thus, our study shows that distinct environmental and biological factors drive bacteria and fungal communities in the wild. This work provides a novel insight into the factors that modulate the rhizosphere microbiome associated with native species in the Ecuadorian *páramo* and sets a baseline for this kind of study in the tropical Andes.

## 2. Materials and Methods

### 2.1. Sample Collection and Processing

**Figure 1:**
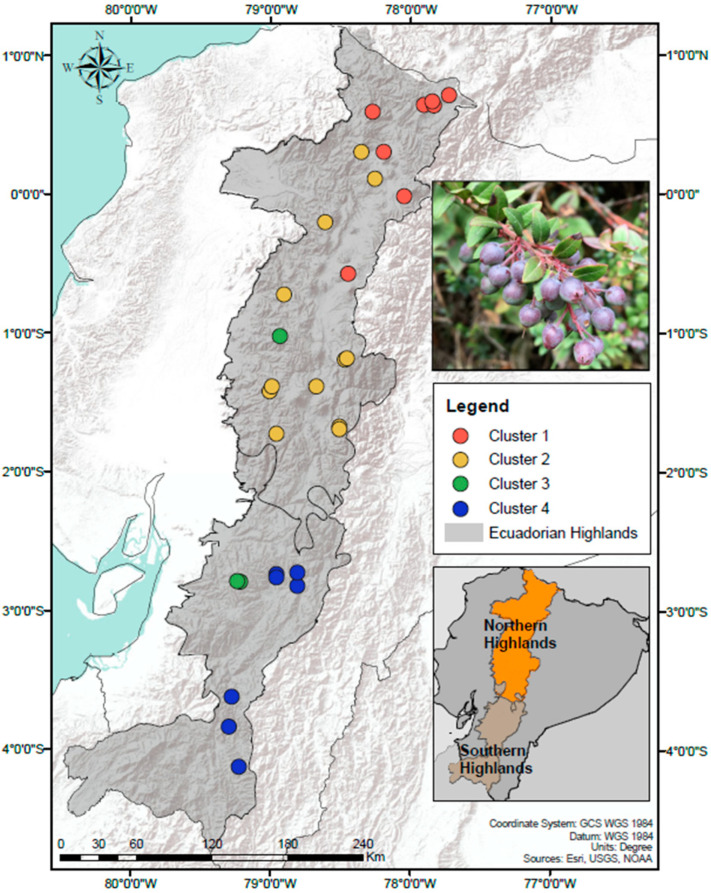
Sampling distribution of *V. floribundum* along the Ecuadorian Highlands.

A total of 39 samples of *V. floribundum*’s soil rhizosphere were selected for this study, based on the results obtained in the previous study of the plant’s genetic diversity [18]. These samples were distributed along the two different soil regions from the Ecuadorian Highlands: northern, and southern [19] (Figure 1). This division is based in the morphological, mineralogical, and physicochemical characteristics of the soil, where northern soils are classified as Andisols (higher nutrient content) while southern soils are Paleosols (poorer soils due to erosion) [19,20].

*V. floribundum* roots were removed from the soil with a shovel. The soil adhered to the roots of each plant (~5 g) was collected by vigorous shaking and placed in Ziploc bags. Samples were transported at 4 °C to the Plant Biotechnology Laboratory at the Universidad San Francisco de Quito, where they were stored at −80 °C until DNA extraction.

DNA extraction was carried out from 0.25 g of soil using the PowerSoil DNA Isolation Kit (MO BIO Laboratories, Carlsbad, CA, USA) following the manufacturer’s instructions. For the amplicon library preparation and sequencing, DNA samples (~200 ng) were sent to the High Throughput Sequencing Facility (HTSF) of the University of North Carolina at Chapel Hill. All PCR procedures and Illumina sequencing MiSeq v3 (2 × 300 bp) (Illumina, San Diego, CA, USA) were performed as previously described by Comeau et al. (2017) [21].

Additionally, 100 g of soil near to the plants was collected for physicochemical analysis. Organic carbon (%), pH, conductivity (µs/cm), total nitrogen (%), Ba (mg/kg), Cd (mg/kg), Co (mg/kg), Cu (mg/kg), Cr (mg/kg), K (mg/kg), Mn (mg/kg), Na (mg/kg), Ni (mg/kg), P (mg/kg), and Pb (mg/kg) were quantified at the Laboratory of Environmental Engineering at the Universidad San Francisco de Quito according to standard procedures [22,23].

### 2.2. Amplicon Sequence Data Processing

Sequences obtained from the 16S rRNA and the ITS rhizosphere libraries were processed separately. Bacterial 16S rRNA sequence reads were processed using MT-Toolbox software [24]. Usable read output from the software (i.e., reads with 100% correct primer and primer sequences that successfully merged with their pair) were quality-filtered using Sickle [25], not allowing any window with a Q score under 20. After the quality control, samples with low total reads recruited (<120,000 reads) were discarded. The resulting sequences were clustered into Amplicon Sequence Variants (ASVs) with the R package DADA2 version 1.8.1 [25]. The taxonomic assignment of each ASV was done using the naïve Bayes *k*-mer method implemented in the DADA2 package using the Silva 132 database as training reference. Fungal ITS forward sequence reads were processed using DADA2 [25] with default parameters. The taxonomic assignment of each ASV was done using the naïve Bayes *k*-mer method implemented in the MOTHUR package [26] using the UNITE database [27] as training reference.

### 2.3. Alpha and Beta Diversity Analyses

Bacterial and fungal count tables were processed and analyzed with the functions of the ohchibi package [28]. Both tables were rarified to 120,000 reads per sample. The diversity function from the vegan package version 2.5-3 [29] was used to calculate an alpha-diversity index (Shannon diversity index). An analysis of variance (ANOVA) was performed to test for differences in the Shannon diversity indices between groups. Tukey’s HSD post hoc tests here and elsewhere were performed using the cld function from the emmeans R package [30]. Beta-diversity was analyzed using the Bray–Curtis dissimilarity calculated from the rarefied abundance tables and visualized through a principal coordinate analysis (PCoA).

Permutational analysis of variance (PERMANOVA) was performed using the adonis function of the vegan package version 2.5-3v [29]. The function chibi.permanova from the ohchibi package [28] was used to plot the R^2^ values for each significant term in the PERMANOVA model tested.

The relative abundance of bacterial phyla and fungal classes was exemplified using the stacked bar representation encoded in the chibi.phylogram function of the ohchibi package [28].

### 2.4. Linear Mixed Models for Alpha-Diversity

To find out the main factors shaping the bacteria alpha diversity, we used an Analysis of Variance (ANOVA). Additionally, sequencing depth (LDepth) and altitude range were included as control variables since they are the most common cofounding factor [8]. We built the next model:

Shannon ~ Soil_region*Plant_genotype*Altitude_range + LDepth

To understand more deeply what specific soil factors impact the Shannon diversity index, we created a first model that included all the edaphic factors measured (Figure S1) plus sequencing depth (LDepth) as a control variable:

Shannon ~ LDepth + pH + log10(OC) + log10(Conductivity) + log10(N) + log10(Co) + log10(Cu) + log10(Cr) + log10(K) + log10(Mn) + log10(Ni) + log10(P) + log10(Pb) + log10(Ba) + log10(Cd) + log10(Na)

Next, we performed variable selection using a stepwise regression algorithm with the function stepAIC [31]. After variable selection, only phosphorous and lead were included in the optimized model.

Shannon ~ LDepth + log10(P) + log10(Pb)

To evaluate the robustness of the model, we transformed the remaining edaphic factors to principal components and incorporated them in the model. We chose then the first three principal components (PC1, PC2, PC3) (Figure S2) that cover up to 80% of the cumulative variance. The final model was:

Shannon ~ LDepth + log10(P) + log10(Pb) + Edsha_PC1 + Edsha_PC2 + Edsha_PC3

## 3. Results

### 3.1. Bacterial Alpha-Diversity Differed across the Soil Regions of the Ecuadorian Highlands

To contrast the effect of abiotic versus biotic factors over the *V. floribundum* rhizosphere microbiome, we first investigated how the soil (i.e., an abiotic factor) modulated the bacterial and fungal communities. Along the Ecuadorian Andes, different types of soils can be found, and their distribution depends on the geology and geography of the Cordillera. Winckell et al. (1997) classified the soils of the Ecuadorian Andes into two distinct regions based on their morphological, mineralogical, and physicochemical characteristics: northern and southern regions [19,20,32].

**Figure 2:**
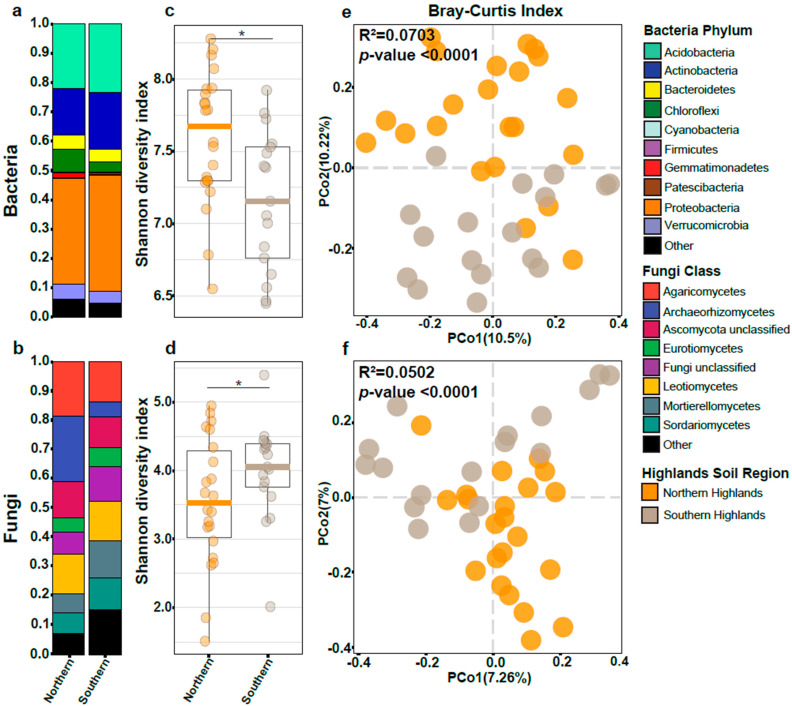
Bacteria and fungi rhizosphere microbiome analysis of the Andean Blueberry from the northern and southern Highlands. Microbiome composition for bacteria (**a**) and fungi (**b**). Boxplot of the entropy (Shannon Diversity Index) for bacteria (**c**) and fungi (**d**). * represents significant differences using *t*-Student test (*p* value < 0.001). Principal Coordinate Analysis based on Bray–Curtis dissimilarities between bacterial (**e**) and fungal (**f**) communities across Highland soil regions. The variance explained (R^2^) and the significance (*p*-value) are shown for a PERMANOVA model.

Using the V3-V4 region of bacterial 16S rRNA, we found that Proteobacteria and Acidobacteria were the most abundant bacterial phyla in all samples regardless of the soil region. The relative abundance of Actinobacteria, Chloroflexi, and Gemmatimonadetes were distinctly different between soil regions (Figure 2a). Fungal communities were characterized by sequencing the internal transcriber spacer (ITS1). Fungi microbiome composition showed a similar distribution across all soil regions, except for the Archaeorhizomycetes and the lower abundant classes group (Figure 2b).

The differences observed in the bacteria and fungi microbiome structure between soil regions were further characterized by assessing the entropy (Shannon Diversity Index). For bacteria, the southern region had the lowest entropy (Figure 2c). Interestingly, for fungi the entropy showed an inverse pattern (Figure 2d).

Community compositional differences between samples were evaluated using the Bray–Curtis dissimilarity index and visualized through a Principal Coordinate Analysis (PCoA). Samples from the northern region were separated from the southern region by the PCo2 axis for bacteria (Figure 2e) and fungi (Figure 2f). Variance explained by soil region was higher in bacteria (R^2^ = 0.0703, *p*-value < 0.000) than in fungi (R^2^ = 0.0502, *p*-value < 1 × 10^−5^). These results clearly suggest that the soil region explains some of the bacterial diversity found in the Andean blueberry rhizosphere but its effect is less strong for the fungal diversity.

### 3.2. Plant Genotype Influences Alpha-Diversity in Fungi

Plant genotype (i.e., biotic factor) has been reported to affect microbiome assembly [7,8], but little of this work was performed in the wild; thus, the question remains as to how strongly plant genotype affects microbiome assembly. To study this, we classified the samples according to the four plant genetic clusters that were previously reported by Vega-Polo et al. (2020) [18] and investigated how the bacterial and fungal community composition varies with plant genotype.

**Figure 3:**
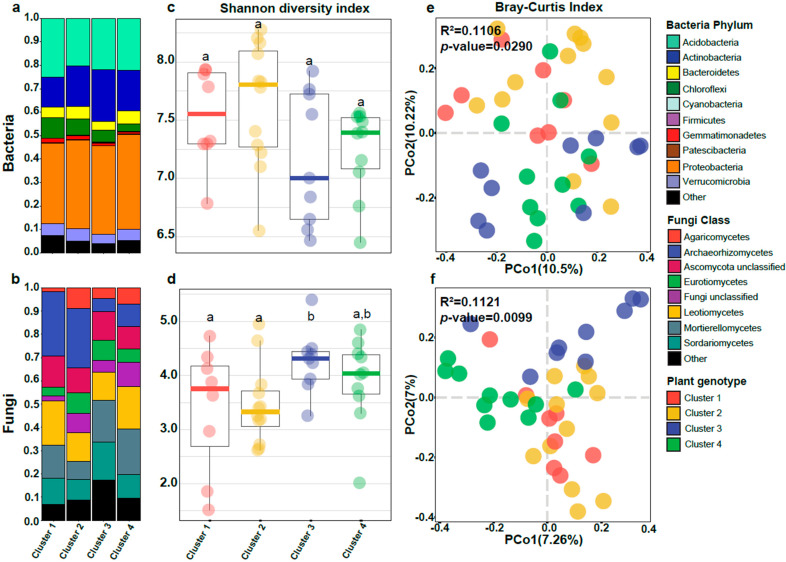
Plant genotype effect over bacterial and fungal communities from the Andean Blueberry rhizosphere. Microbiome composition for bacteria (**a**) and fungi (**b**). Boxplot of the entropy (Shannon Diversity Index) for bacteria (**c**) and fungi (**d**). Letters represent the results of the Tukey HSD post hoc test for one-way ANOVA. Principal Coordinate Analysis based on Bray–Curtis dissimilarities between bacterial (**e**) and fungal (**f**) communities across plant genotype clusters. The variance explained (R^2^) and the significance (*p*-value) are shown for a PERMANOVA model.

Contrary to what was found for the soil region, the bacterial communities did not show substantial differences between plant genetic clusters (Figure 3a). In turn, fungal communities from Cluster 3 and 4 displayed a distinct class composition compared to the other two clusters, with a reduced abundance of Archaeorhizomycetes and an increase of Mortierellomycetes (Figure 3b). This suggests that plant genotype plays more of a role in defining fungal community than bacterial.

For alpha diversity, the Shannon diversity index only showed significant differences for fungi (Figure 3c,d), showing that plant genetic clusters 3 and 4 present the highest entropy compared to the other two.

Beta-diversity was analyzed using the Bray–Curtis dissimilarity index. Variance explained by plant genotype was similar for bacteria (R^2^ = 0.1106, *p*-value = 0.0290) and fungi (R^2^ = 0.1121, *p*-value = 0.0099). Clusters 1 and 2 did not exhibit any separation in the PCoA for bacteria and fungi (Figure 2e,f). Moreover, neither cluster had significant differences for alpha diversity. Cluster 3 was distributed in two clear groups for bacteria in the PCoA (Figure 3e), which is correlated with the bimodal distribution observed in Shannon diversity (Figure 3c). Cluster 3 and Cluster 4 were clearly separated only for fungi but not for bacteria. These results hint that plant genotype could have a stronger effect over fungal communities than bacterial in the *V. floribundum* rhizosphere.

### 3.3. Soil Region Is the Main Predictor for Alpha-Diversity of Bacterial Communities, While Fungal Diversity Is Driven by the Interaction between Plant Genotype and Altitude

To summarize the effect of abiotic factors (e.g., soil region) and biotic factors (e.g., plant genotype) over alpha diversity, we used an analysis of variance to identify the main effects and interactions. For bacteria, soil region had the higher F values for the Shannon diversity index, making it the main predictor (Table 1). Conversely, the interaction between plant genotype and altitude is the main predictor for fungal communities while soil region has a barely significant effect on fungal diversity.

### 3.4. Bacterial Alpha-Diversity Is Correlated with the Phosphorus and Lead Found in the Páramo Soils

**Figure 4:**
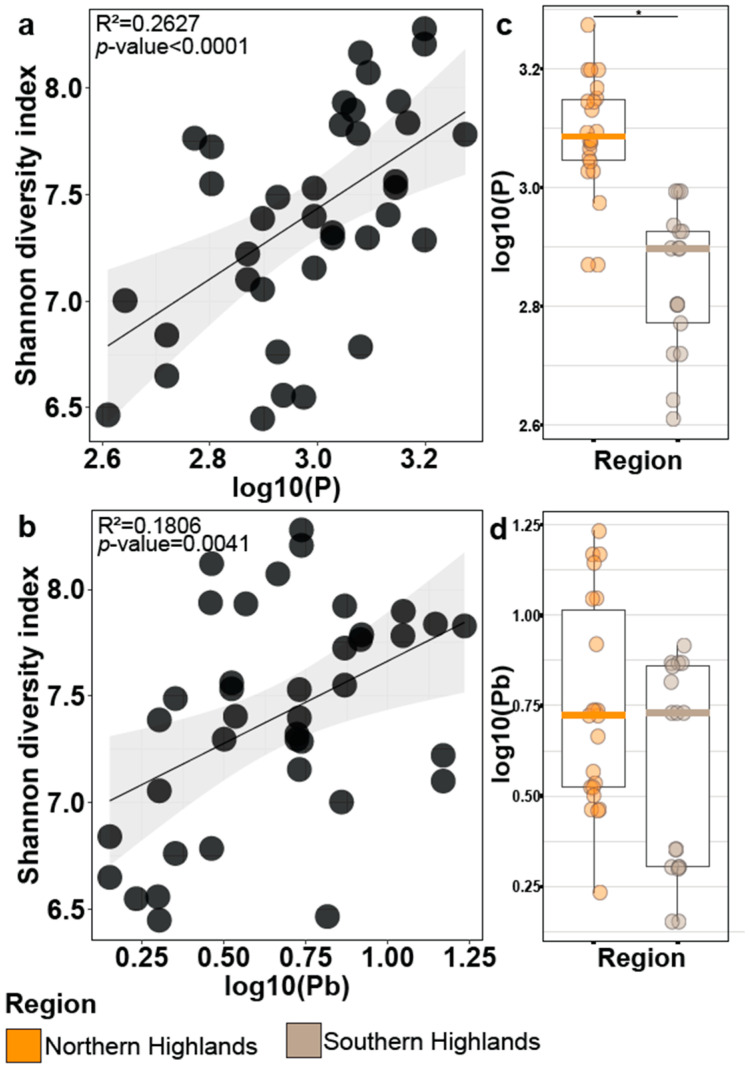
Phosphorous and lead drive relationship between Shannon diversity index of the bacterial rhizosphere and soil regions. Dispersion graph and regression analysis of Shannon diversity index versus phosphorous (**a**) and lead (**b**). Regression coefficient (R^2^) and the significance (*p*-value) are presented for each factor. Boxplot of phosphorous (**c**) and lead (**d**) concentration found in the three soil regions. * represents significant differences using *t*-Student test (*p*-value < 0.001).

As soil region is the main factor influencing bacterial alpha diversity in the rhizosphere, we investigated what edaphic factors may be involved and could explain this diversity. We measured 15 factors (See Methods, Figure S1) from bulk soil. We performed variable selection using a stepwise regression algorithm [31] to determine which factors in the soil best explained the Shannon diversity index from bacteria. The best statistical model included only two factors: phosphorous (P) and lead (Pb). Combined, both factors explained up to 40.65% of the Shannon diversity index (Table 2). When analyzed individually, P had a higher regression coefficient (R^2^ = 0.2627 *p*-value < 1 × 10^−5^) than Pb (R^2^ = 0.1806 *p*-value = 0.0041) (Figure 4a,b). Finally, we tested the influence of the other edaphic factors; the variance explained by the full model increased by less than 1% (Table 2). Thus, P and Pb are sufficient to explain the patterns of this diversity index, when considering the other edaphic factors analyzed in the bulk soil.

To quantify the relation between the soil regions and the edaphic factors, we examined the concentrations of P and Pb (Figure 4c,d). We found significant differences between soil regions for P; the pattern was similar to that observed for alpha diversity in bacteria (Figure 2c). The north region that had higher P concentration also yielded a higher entropy for bacteria; commensurately, the south region held the lowest P concentration and lowest alpha diversity.

## 4. Discussion

In this study, we use the soil differences in the Ecuadorian Highlands and the population structure of *V. floribundum* to assess how edaphic factors and plant genotype influence the assembly of bacterial and fungal communities in the rhizosphere. Our results show that bacteria and fungi responded differently to these two factors in this natural setting. Bacterial community composition is mainly predicted by soil region and its associated edaphic factors, specifically P and Pb. In contrast, fungal communities are relatively strongly influenced by the interaction between plant genotype and altitude.

Soils are complex mixtures of minerals, water, air, organic matter, and organisms, both living and dead. Soils are also the primary source of microorganisms that serve as inoculum to colonize the rhizosphere and plant tissues [33]. Soil microbial communities are diverse and heterogeneous mainly due to the edaphic factors and environmental conditions [34]. In Ecuador, volcanic activity in the northern part of the country has a substantial effect on soil. In this area, deep Andisols with a high organic carbon concentration have developed. High nutrient content in these soils is maintained thanks to the minerals being renewed from ash and lapilli [20]. In turn, the southern part does not have recent superficial pyroclastic accumulation, and its soils are primally formed by rock weathering influenced by the climatic changes in the zone. This phenomenon has produced poor ferritic Paleosols and Vertisols that are often degraded by erosion and have a shallow organic layer [19]. This natural difference in nutrient content between the soils from the north and south regions could influence the microbiome composition, according to the oligotrophic–copiotrophic theory [34,35]. This theory states that copiotrophic bacterial taxa (e.g., Proteobacteria and Firmicutes) usually are more prevalent in nutrient-rich conditions [34,36,37]; in contrast, oligotrophic bacteria (e.g., Acidobacteria, Gemmatimonadetes, Verrucomicrobia, and Chloroflexi) can maintain growth and be prevalent under nutrient-poor conditions [37,38,39]. We hypothesized that these differences in the soil nutrient content could be influencing partially the bacterial composition in the rhizosphere of the *V. floribundum* in Ecuador.

Our results showed that Proteobacteria and Acidobacteria were the most abundant bacterial phyla found in the rhizosphere soil samples associated with the Andean blueberry (Figure 2a). These findings are not surprising, because Proteobacteria members are well adapted to plant rhizospheres across diverse plant species [1,40], and Acidobacteria is one of the most abundant bacterial taxa in soils, especially in acidic conditions [7,41]. Additionally, Proteobacteria and Acidobacteria are the most dominant bacterial phyla in the rhizosphere microbiomes of other *Vaccinium* species [5,42,43]. Contrary to our hypothesis and as predicted by the oligotrophic–copiotrophic theory, we found that the relative abundance of Proteobacteria increased while the Verrucumicrobia decreased from north to south (Figure 2a). Only Acidobacteria show an increment in the south region, and they are related to nutrient-poor soils [34,41] such as those in the southern Ecuadorian region [19]. Additionally, we also found that the copiotrophic phylum Firmicutes and oligotrophic phyla Gemmatimonadetes and Chloroflexi did not follow the predicted patterns. These results point out that more factors, not only soil nutrient content, affect the community composition of the *V. floribumdum* rhizosphere.

Plants can change the composition and structure of the microbiome by secreting substances to modulate root microorganisms according to their needs [1]. Consequently, bacteria initially present in the soil respond differently to the rhizosphere environment, experiencing a change in their abundance or even disappearing [6,44]. However plausible, there is currently no direct evidence that root metabolites shape the microbiome composition of the Andean blueberry.

Given that soil is an amalgam of diverse materials, we sought to understand if abiotic factors such as mineral content were driving the relationship between soil and bacterial communities. We used a stepwise variable selection algorithm to identify key edaphic factors that could explain bacteria alpha diversity. We found that P and Pb alone explained most of the Shannon Diversity Index variance (40.65%) (Table 2), which is consistent with the relationship between P and plant microbiome previously shown using bacterial synthetic communities (Syncoms) and *Arabidopsis thaliana* in the lab [45,46,47].

Mechanistically, the relationship between P and the community makes sense. Castrillo et al. (2017) showed that plant genes *phr1;phl1* and *phf1,* involved in the plant phosphate starvation response (PSR), coordinate microbiome assembly and plant immunity (40). Additionally, Finkel et al. (2019) demonstrated that root bacterial alpha diversity changed proportionally to a P concentration gradient in an agar matrix [46]. Plants exposed to a higher P content had a higher root Shannon Diversity Index. Our results demonstrated that the same effect on the root alpha diversity could be observed for a native species growing in a natural P-gradient (Figure 4a). The effect of Pb on microbiome diversity has not been extensively explored but is likely important. Soil contamination with Pb occurs due to natural and anthropogenic activities, and it influences the richness, diversity, and structure of bacterial communities [48,49]. It has been reported that the bacterial phyla Proteobacteria and Acidobacteria, found as the most abundant phyla in our study (Figure 2a), are predominantly presented in Pb-containing soils [50].

In contrast to many bacterial populations, when we analyzed the fungal communities, we did not observe a clear relation of the fungal diversity with nutrient content across soil regions (Figure 2d). Relationships between fungal communities and soil nutrients have not been widely explored. Prior investigations have observed that soil characteristics correlate weakly with fungal community structure [51,52,53]. Accordingly, the Andean blueberry fungal communities were not dominated by any specific taxa (Figure 2b). Even though the oligotrophic–copiotrophic theory is not commonly used to describe fungi taxa [54], we used this framework to assess the rhizosphere fungal composition of *V. floribundum* among soil regions. Archaeorhizomycetes displayed a decrement from north to south (Figure 2b), matching the behavior of copiotrophic taxa. Saprotrophic fungi have been associated with copiotrophic conditions, plant-litter decomposition, and nutrient cycling [55,56]. *Archaeorhizomyces* is the only genus described for the Archaeorhizomycetes class, and it is associated with the roots of woody species, cellulose degradation, and carbon cycling [57], showing that oligotrophic–copiotrophic theory could, at best, explain only a small fraction of fungi microbiome composition. Even further, our results illustrate how bacterial communities are highly affected by the soil (abiotic factor), while fungal communities are more stable to soil variations. This effect is also found in other studies that indicate that abiotic factors are more important for bacterial diversity compared to fungal diversity in various ecosystems [58,59].

The earliest plant fossils harbored structures morphologically similar to fungal symbiotic structures, and more than 80% of living plants are symbiotic with fungi [60,61], including associations with specific genotypes [62,63,64]. This close link between host and fungus suggested that plant genotype might shape the fungal composition of the *V. floribundum* microbiome. As noted, *V. floribundum* has four genetic clusters, where individuals from clusters 1 and 2 showed an overlapping distribution within the northern soil region, while individuals from Cluster 3 were distributed on the central and southern regions [18]. Fungal communities displayed clear differences among plant genetic clusters with three groups formed in the PCoA (Figure 3f). The first group represented rhizosphere samples coming from plant genetic clusters 1 and 2, while samples from plant genetic clusters 3 and 4 were separated into two independent groups.

Interestingly, in the linear mixed model used to explain alpha diversity (Table 1), plant genotype was barely significant (F-value = 2.814, *p*-value = 0.0561), yet the interaction between plant genotype and altitude had the highest F-value (F-value = 9.652 and *p*-value = 0.0036). This result is related to the findings of Vega et al. (2019) on the *V. floribundum* genetic diversity and genetic distribution, which showed that the expected plant heterozygosity decreases while the elevation increased and that Cluster 4 is restricted to higher altitudes, making the individuals of this cluster part of a “sky island” with a reduced genetic flow to other locations [18]. Although altitude has been reported to be a significant factor that affects the fungal composition in the soil and the rhizosphere, its effect is mainly indirect by driving the distribution of plant species [65,66,67]. Altogether, our results indicate that bacterial and fungal communities in the rhizosphere respond differently to the plant genetics in the wild, which is consistent with earlier work suggesting a link between plant community composition and fungal diversity [68,69]. We believe this is because fungal communities depend more greatly on biotic factors (host-specific factors) than bacterial communities, which are more shaped by the edaphic factors of the soil.

Here we show that abiotic and biotic factors shape the bacterial and fungal communities in the *V. floribundum* rhizosphere in fundamentally different ways. We found that soil, especially as defined by edaphic factors such as P and Pb gradients, and its geological history are key to bacteria microbiome assembly in the *páramo*. In contrast, host genotype, a biotic factor, was critical for defining fungal communities. Our data is, of course, from one host species in one part of the world. It is vital to expand the microbiome study to other plant species that are not cultivated or domesticated and make use of the environmental variation to test different hypotheses in natural experiments and to understand the interplay of soil and genotype in defining microbiome assembly more broadly. This work will also help us better understand the diversification and radiation in the Ecuadorian Highlands so that we can align it with conservation and domestication aims.

## Figures and Tables

**Table 1:** Factors predicting alfa-diversity of rhizosphere bacterial and fungal communities.

	Shannon Diversity Index	
	Bacteria	Fungi
	R^2^ = 0.3623 (0.0058)	R^2^ = 0.4694 (0.0155)
Region	**7.857 (0.0088)**	3.581 (0.0681)
Plant genotype	0.096 (0.9618)	2.814 (0.0561)
Altitude range	0.316 (0.7314)	0.195 (0.8242)
LDepth	1.875 (0.1811)	2.159 (0.1521)
Plant genotype × Altitude range	2.181 (0.1501)	**9.652 (0.0036)**

F-value is presented with *p*-value between parentheses.

**Table 2:** Main edaphic factors predicting the Shannon diversity index of rhizosphere bacterial communities.

	Shannon Diversity Index
	R^2^ = 0.4137 (0.0005)
log_10_P	**2.213 (0.0341)**
log_10_Pb	**2.105 (0.0432)**
Edaphic factor PC1	0.176 (0.8614)
Edaphic factor PC2	1.322 (0.1957)
Edaphic factor PC3	0.237 (0.8144)
only log_10_P + log_10_Pb	**R^2^ = 0.4065 (3.15 × 10^−5^)**

F-value is presented with *p*-value between parentheses.

## Data Availability

Genetic data for specimens were obtained under the Genetic Resources Permit Number: MAE-DNB-CM-2016-0046 granted to Universidad San Francisco de Quito by Ministerio del Ambiente Ecuador, in accordance with the Ecuadorian law. Amplicon sequences from 16s and ITS regions are archived in the National Center for Biotechnology Information SRA database (BioProject accession no.: PRJNA769071). The ASV tables, and experimental data are archived and publicly available on Github (https://github.com/darioxr/rhizosphere_Vfloribundum) (accessed on 4 January 2023).

## Supplementary Materials

The following supporting information can be downloaded at: https://www.mdpi.com/article/10.3390/microorganisms11020399/s1, Figure S1: Correlation analysis between edaphic factors; Figure S2: Principal component analysis and contributions from edaphic factors after removing phosphorous and lead.
