# Supplementary material for: Untangling the Effects of Plant Genotype and Soil Conditions on the Assembly of Bacterial and Fungal Communities in the Rhizosphere of the Wild Andean Blueberry (Vaccinium floribundum Kunth)
Source: Microorganisms. 2023 Feb 4;11(2):399. doi: 10.3390/microorganisms11020399 (PMC9961955; doi:10.3390/microorganisms11020399)
Supplement: Supplementary file 1 [file microorganisms-11-00399-s001.zip › microorganisms-2146986-supplementary.pdf]

SUPPLEMENTAL FIGURES

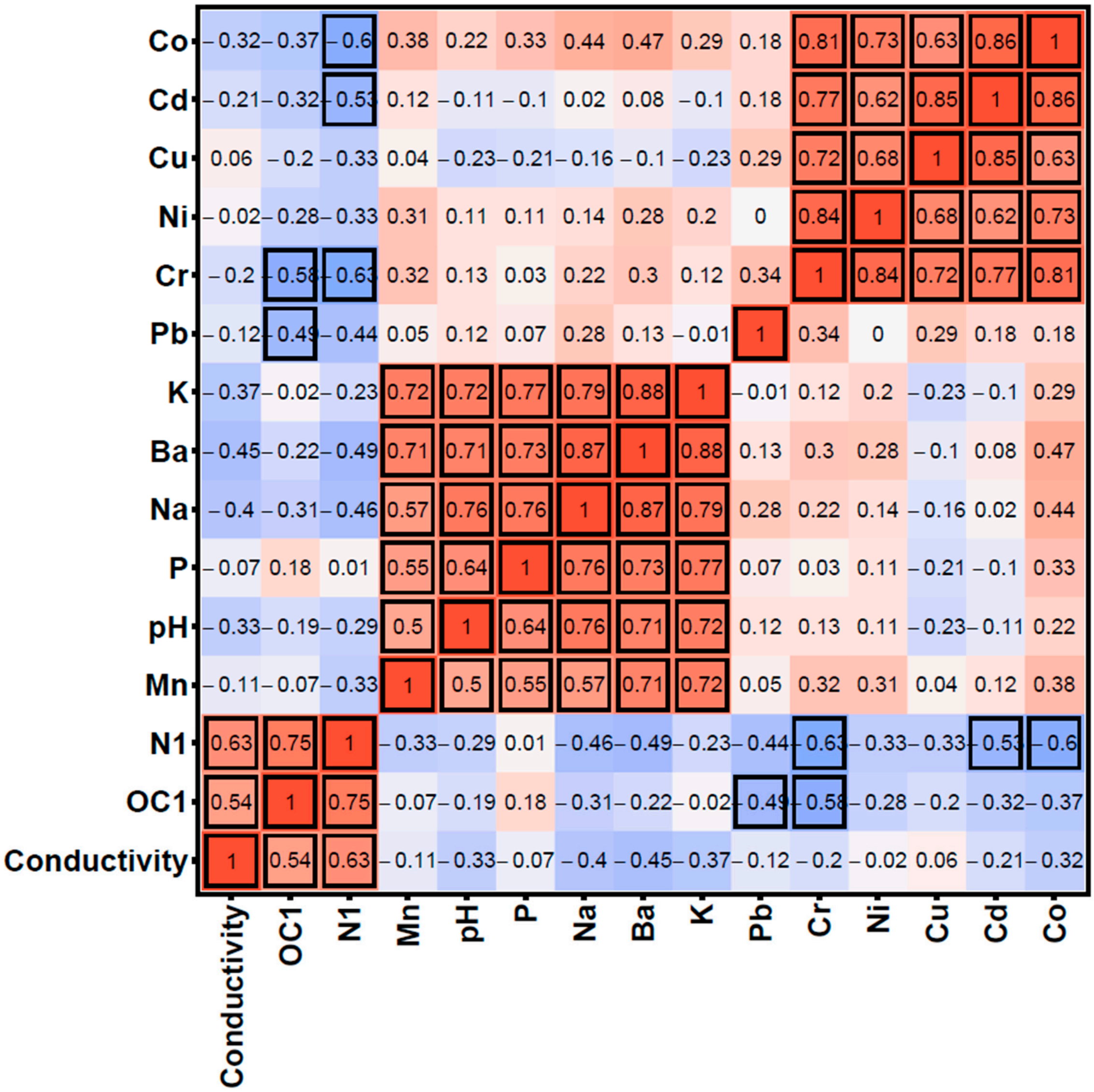

Figure S1. Correlation analysis between edaphic factors. Pearson correlation coefficient is showed. Title border indicates *p*-value < 0.05.

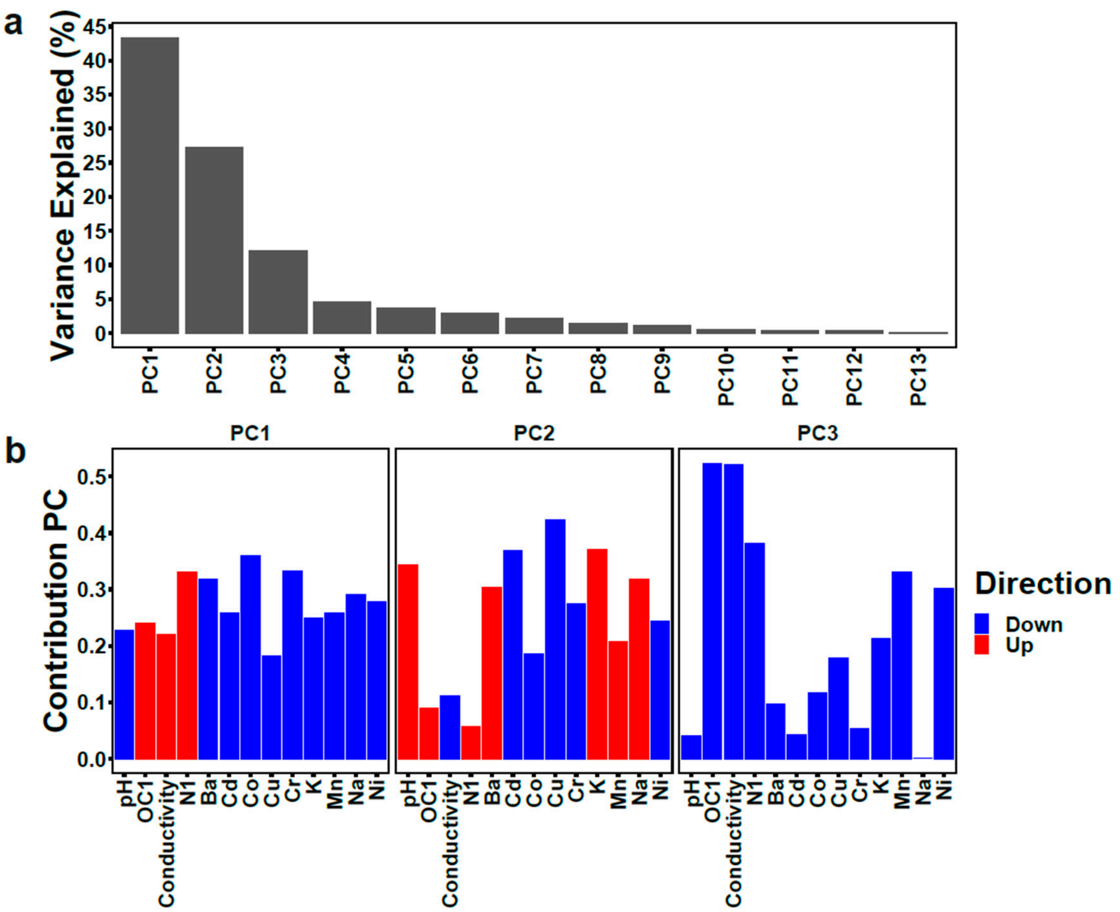

Figure S2. Principal component analysis and contributions from edaphic factors after removing phosphorous and lead. Upper panel (a) show the variance explain for each component. The lower panel (b) show the induvial contribution of each edaphic factor to the first three components.
